# Unexpected course of reaction between (*E*)-2-aryl-1-cyano-1-nitroethenes and diazafluorene: why is there no 1,3-dipolar cycloaddition?

**DOI:** 10.1007/s00706-016-1893-5

**Published:** 2017-03-22

**Authors:** Radomir Jasiński, Karolina Kula, Agnieszka Kącka, Barbara Mirosław

**Affiliations:** 1 Institute of Organic Chemistry and Technology, Cracow University of Technology, Warszawska 24, 31-155 Cracow, Poland; 2 Department of Crystallography, Maria Curie-Skłodowska University, Maria Curie-Skłodowska 3, 20-031 Lublin, Poland

**Keywords:** Alkenes, Cycloadditions, Diazo compounds, Quantum chemical calculations, Reaction mechanism

## Abstract

**Abstract:**

Reactions between (*E*)-2-aryl-1-cyano-1-nitroethenes and diazafluorene lead to acyclic 2,3-diazabuta-1,3-diene derivatives, instead of the expected pyrazoline systems. DFT calculations suggest that this is a consequence of formation of zwitterionic structure in the first stage of the reaction. It must be noted that this is a specific property of the (*E*)-2-aryl-1-cyano-1-nitroethenes group, in contrast to most other conjugated nitroalkenes.

**Graphical abstract:**

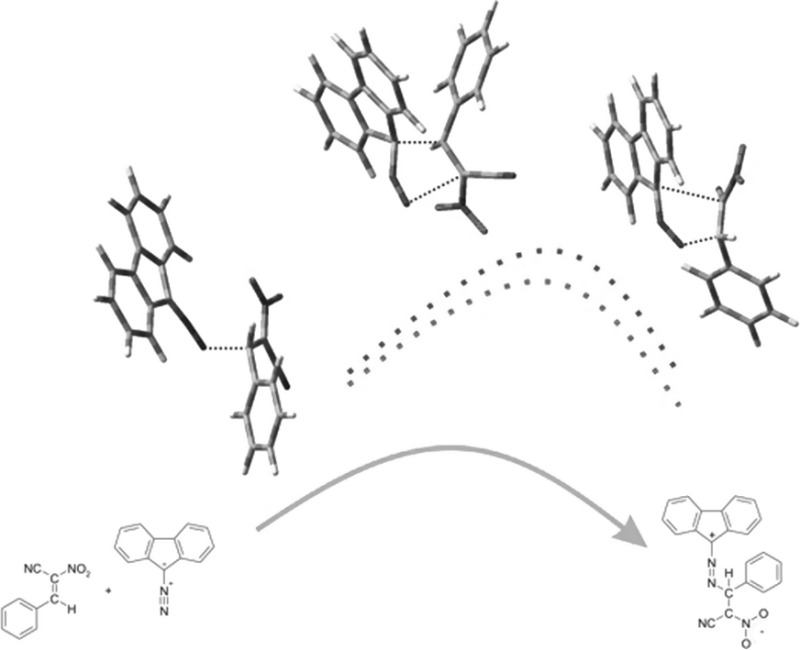

**Electronic supplementary material:**

The online version of this article (doi:10.1007/s00706-016-1893-5) contains supplementary material, which is available to authorized users.

## Introduction

Conjugated nitroalkenes (CNA) are very useful and universal synthons in organic synthesis. On their basis, many valuable compounds may be prepared, such as nitronic acid esters [1], amines [2], oximes [3] and others [2]. Additionally, the presence of a highly electron-withdrawing nitro group stimulates π-deficiency of a double bond, which activates these compounds in a stereo-controlled reaction with nucleophilic reagents such as dienes [2, 4, 5], 1,3-dipoles [3, 6] and acetylenes [4].

(*E*)-2-Phenyl-1-cyano-1-nitroethene and their aryl-substituted analogs (ACNE) were prepared for the first time in the first half of the twentieth century [7]. At present, several compounds from this group are known [8–11]. However, their chemical properties are not well known. Some compounds have been described very recently. In particular, some examples of participation of ACNE in Diels–Alder reactions as dienophiles [12–15] as well as heterodienes [8, 16, 17] are explored. Additionally, some examples of catalyzed 1,3-dipolar cycloadditions between ACNE and trimethylsilyl azide are also analyzed [18, 19]. Unfortunately, this method does not yield stable adducts, because the primary reaction products decompose partially under reaction conditions. Actually, any examples of thermal (non-catalyzed) 1,3-dipolar cycloadditions involving ACNE are unknown. This work is an attempt to fill this gap and is a continuation of our systematic study about participation of CNA in cycloaddition reactions [15, 20–24]. In particular, we have decided to shed light on the reactions between the homogenous series of ACNE (**1a**–**1d**) and diazafluorene (**2**), as a model allenyl-type 1,3-dipole with >(C^−^)–(N^+^)≡N functional group. Theoretically, these reactions should give attractive, from a practical point of view, nitrofunctionalized pyrazoline systems (Scheme 1). In addition to experimental studies, we also performed comprehensive quantum chemical studies to understand better the nature and molecular mechanism of these reactions.

**Figure:**
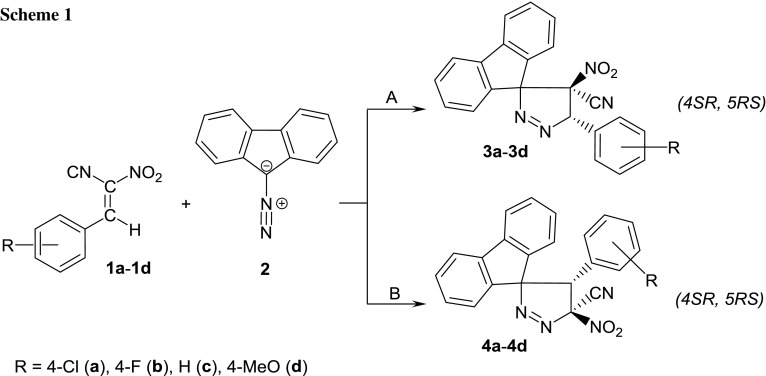

## Results and discussion

Firstly, we prepared the reaction components. For this purpose, we applied methodologies described earlier (see the experimental section). Next, the analysis of the interaction between the addends in the expected cycloaddition course was performed. Global and local electronic properties of (*E*)-2-aryl-1-cyano-1-nitroethenes had been analyzed in detail previously [17, 25]. It was discovered that all these compounds were characterized by a high global electrophilicity (in the case of compounds **1a**–**1d** and were in the range of 3.14−3.68eV). In comparison (Table 1), diazafluorene has evidently weaker electrophilic nature (ω = 1.84 eV). Additionally, **2** is characterized by a relatively high global nucleophilcity (more than 3.5 eV). In consequence, the interaction between CNA **1a**–**1d** and **2** may be considered a polar one [26]. Subsequently, the local electronic properties of addends have been analyzed. As established earlier, in all CNA **1a**–**1d**, most of the electrophilic center is located at the β-position of the nitrovinyl moiety [17, 25]. On the other hand, most of the nucleophilic center in the >CNN moiety of **2** is located on the terminal nitrogen atom. In polar cycloadditions, a reaction course is controlled by the nature of the local nucleophile–electrophile interaction. Therefore, we have assumed that the reaction channel A should be preferred.

**Table 1:** Selected global and local electronic properties for diazafluorene (2) in comparison to other diazocompounds

Diazocompound	Global properties		Local properties			
	ω/eV	*N*/eV	*P*_C_^−^	*P*_N_^−^	*N*_C_/eV	*N*_N_/eV
Diazafluorene (**2**)	1.84	3.61	0.23	0.39	0.83	1.41
4-Methylphenyl-phenyl-diazomethane [27]	1.47	4.00	0.21	0.41	0.86	1.63
4-Chlorophenyl-phenyl-diazomethane [27]	1.70	3.75	0.22	0.40	0.81	1.50

**Fig. 1:**
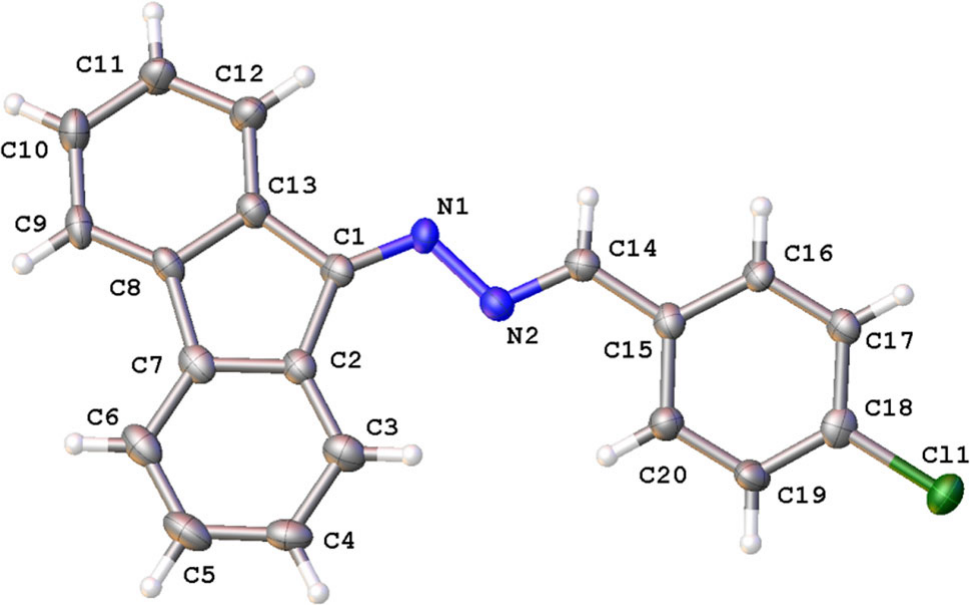
Molecular structure of** 5a** with atom labels

To verify the quantum chemical simulations, we performed experimental tests of the reactions of interest. In the first step, we explored reactions involving ACNE **1a**. It was found that this reaction proceeds in MeNO_2_ solution under mild conditions and yields a dark brown solid. HPLC analysis of the post-reaction mixture shows the existence of one reaction product, which was isolated by crystallization from ethanol. Its constitution was established by means of elemental analysis as well as spectral techniques. It was found unexpectedly that the results of the elemental analysis were fundamentally different from those of the expected adduct. Next, in the IR spectrum, any bands from NO_2_ as well as CN groups were not observed. On the other hand, the MS spectrum gives a molecular ion, which suggests that the molecular weight is less than that in the expected adduct. This suggest the absence of the C(CN)NO_2_ moiety in the molecule. Thus, we established that it is not a five-membered heterocyclic product, but a 2,3-diazabuta-1,3-diene derivative **5a**. This was fully confirmed by the single crystal X-ray structure determination (Fig. 1 as well as Supplementary Material). For a full characteristic of this compound, ^1^H and ^13^C NMR spectra were also recorded (see “Experimental” section).

Similarly, we analyzed the reactions involving ACNE **1a**–**1d**. In all cases, respectively, 2,3-diazabuta-1,3-diene derivatives **5b**–**5d** were isolated instead of the expected nitropyrazolines. Next, we tested the reactions between diazafluorene **2** and used ACNE in other, different solvents such as acetonitrile, DCM and chlorobenzene. In all cases, only 2,3-diazabuta-1,3-diene derivatives were isolated, without any cycloadducts.

This phenomenon can be explained when assuming that the primary reaction product **3a**–**3d** spontaneously decomposed with: C(CN)NO_2_ carbene elimination according to the retro-[4 + 1]-cycloaddition scheme (path C on Scheme 2). Theoretically, the highly substituted five-member heterocycles may have decomposed with the ring opening via carbene elimination. It should be highlighted, however, that any cases of this type of decomposition of nitropyrazoline systems had not been previously described. Some of these heterocycles decomposed under mild conditions, but via completely different mechanisms [28–32]. Alternatively, it may be assumed that in the first reaction stage, a zwitterionic intermediate **I** is formed. The zwitterionic structure of **I** is probably stabilized by a push–pull electronic effect, which is determined by the presence of two EWG groups (NO_2_ and CN) in the terminal position. A similar effect has been recently explored [11] in detail in the case of a molecule, which has similar structural moieties. In the next step, it is converted via C(CN)NO_2_ carbene elimination into a 2,3-diazabuta-1,3-diene derivative (Scheme 2). It should be noted at this point that the possibility of the existence of zwitterionic structures on the paths of reactions involving CNA has been recently described in the case of interactions between diarylnitrones and 1-EWG-substituted 1-nitroethenes [33, 34] as well as between thiocarbonylylides and nitroethene [35, 36].

**Figure:**
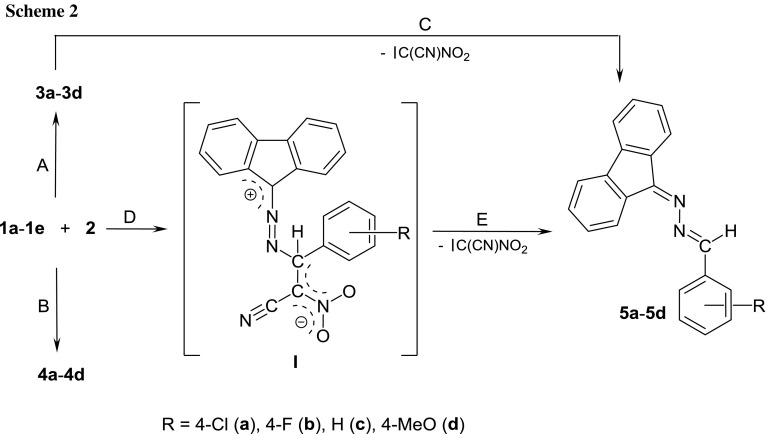

**Fig. 2:**
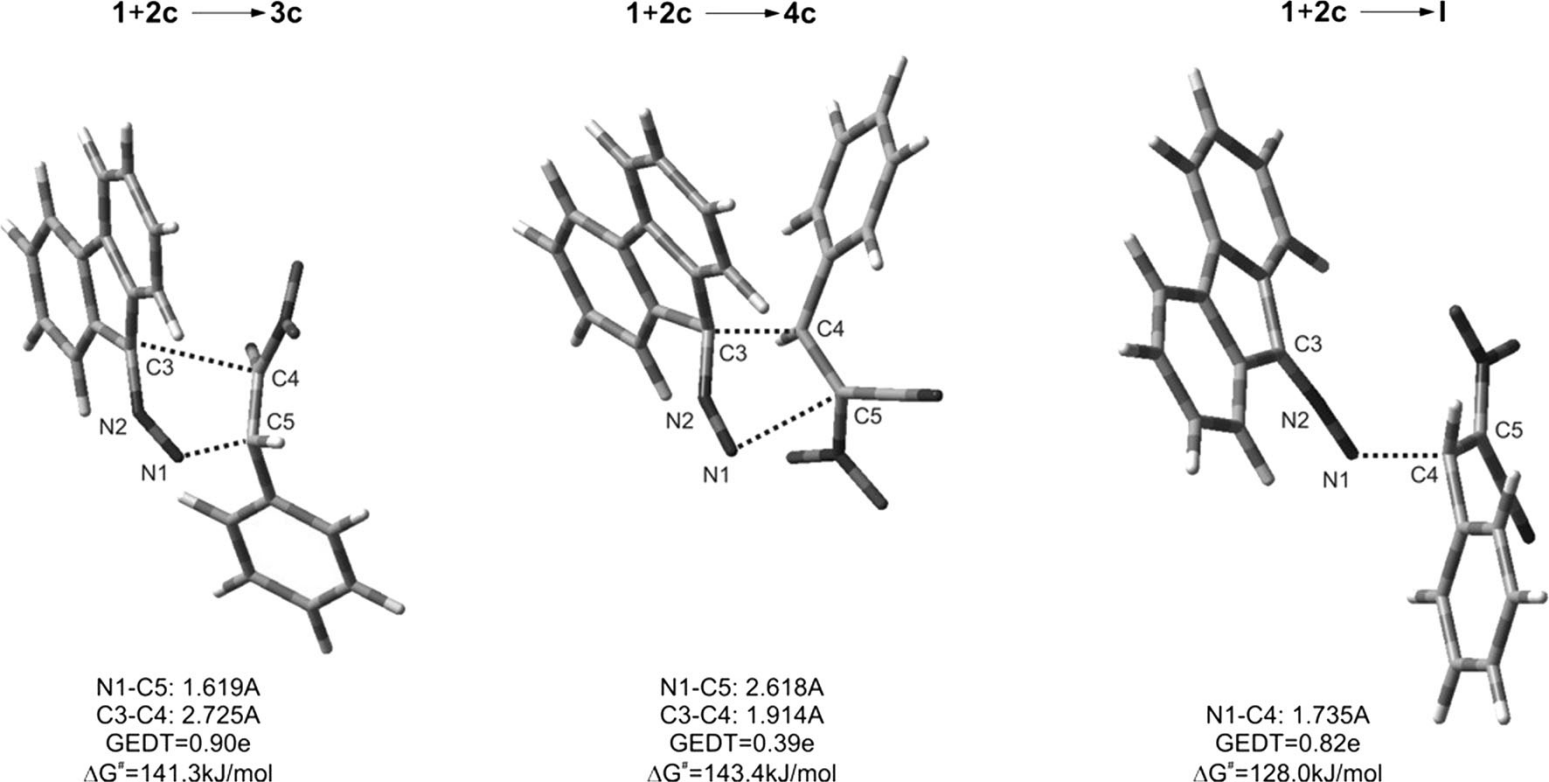
Views of TSs for reaction between** 1c** and** 2**

To confirm this hypothesis, we have performed DFT simulation of theoretically possible paths for a model reaction involving CNA **1c**. It was found that, in contrast to the reaction **1c** + **2** → **I**, both cycloadditions leading finally to pyrazoline systems (**3c** and **4c**) should be treated as forbidden from a kinetic point of view. In particular, for reactions **1c** + **2** → **3c**(**4c**), Gibbs free energy of activation is higher than 141 kJ/mol, whereas in reaction **1c** + **2** → **I** about 128 kJ/mol. In consequence, our calculations may support mechanism via paths D + E, which is illustrated in Scheme 2. The key physical parameters of all considered transition states are presented in Fig. 2. It should be noted that the zwitterionic nature of **I**, as well as the charge distribution on its molecule, was confirmed by a population Mulliken analysis (see the GEDT value).

Finally, it should be noted that the described reaction course is a specific property of the ACNE group. In comparison, a similar reaction between (*E*)-3,3,3-trichloro-1-nitroprop-1-ene (**6**) (which has similar electrophilicity to ACNE [22]) and diazafluorene proceeds in MeNO_2_ solution under mild conditions and gives pyrazoline **7** with quantitative yield (Scheme 3). Interestingly, this adduct is easily decomposed into **8** under relatively mild conditions. This decomposition proceeded via the HCl elimination stage.

**Figure:**
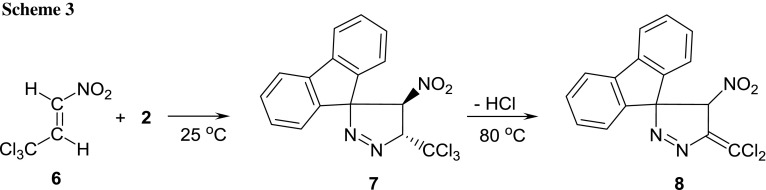

## Conclusion

Reactions between allenyl-type 1,3 dipoles and ethylene/acetylene derivatives proceed generally according to the cycloaddition scheme [27–39]. Unexpectedly, diazafluorene reacts with (*E*)-2-aryl-1-cyano-1-nitroethenes via another mechanism. In particular, in these processes, acyclic 2,3-diazabuta-1,3-diene derivatives are formed instead of heterocyclic adducts. Probably, this is a consequence of the formation of the zwitterionic structure in the first reaction stage. This hypothesis is supported by DFT calculations. It must be noted that there is a specific property of the (*E*)-2-aryl-1-cyano-1-nitroethenes group, in contrast to most other conjugated nitroalkenes. We found that a similar reaction between (*E*)-3,3,3-trichloro-1-nitroprop-1-ene and diazafluorene proceeds under mild conditions and gives 4-nitropyrazoline as the product.

## Experimental

The melting points were determined on a Boetius apparatus and are uncorrected. Elemental analyses were performed on a Perkin-Elmer PE-2400 CHN apparatus. IR spectra were recorded on a Bio-Rad spectrophotometer in CCl_4_ solution. The ^1^H NMR (500 MHz) and ^13^C NMR (125 MHz) spectra were recorded on a Bruker AMX 500 spectrometer. Liquid chromatography (HPLC) was done using a Knauer apparatus equipped with a UV/Vis detector. For monitoring of the reaction progress, LiChrospher 18-RP 5 μm column (4 × 240 mm) and 75% methanol as the eluent at a flow rate of 1.0 cm^3^/min were used.

Nitroalkenes **1a**–**1d** [9, 11] and **6** [46] and diazafluorene (**2**) [45] were synthesized according to the procedures described earlier.

### X-ray crystal structure determination

The X-ray diffraction intensities for **5a** were collected at 120 K on SuperNova X-ray diffractometer equipped with Atlas S2 CCD detector using the mirror-monochromatized CuK_α_ radiation (*λ* = 1.54184 Å). All data were collected using the *ω* scan technique, with an angular scan width of 1.0°. The programs CrysAlis CCD, CrysAlis Red and CrysAlisPro [40, 41] were used for data collection, cell refinement and data reduction. The structures were solved by direct methods using SHELXS-97 and refined by the full-matrix least squares on *F* ^2^ using the SHELXL-97 [42]. The H atoms were positioned geometrically and allowed to ride on their parent atoms, with *U*_iso_(H) = 1.2 *U*_eq_(C). The molecular plot was drawn with Olex2 [43]. Compound **5a** crystallizes in an orthorhombic *Pna*2_1_ space group. The molecule is nearly planar. The two aromatic parts of the molecule are twisted around the central linear fragment by ca. 14.6(9)°. The bond lengths in the linear fragment were as follows: C1 = N1 1.302(6) Å, N1–N2 1.392(6) Å, N2 = C14 1.298(6) Å, and C14–C15 1.442(7) Å, showing some degree of electron delocalization along this molecular fragment in comparison to another related structure of *p*-methoxybenzaldehyde 9-fluorenylidenehydrazone with the respective bond lengths: C8 = N2 1.286 (4) Å, N1–N2 1.418 (3) Å, N1 = C7 1. 264 (4) Å, and C7–C6 1.453 (4) Å (atom labels as in the original paper) [44].

The experimental details and final atomic parameters for 5a have been deposited with the Cambridge Crystallographic Data Centre as supplementary material (CCDC ID 1448932). Copies of the data can be obtained free of charge on request via http://www.ccdc.cam.ac.uk/conts/retrieving.html (or from the Cambridge Crystallographic Data Centre, 12, Union Road, Cambridge CB2 1EZ, UK; fax: +44 1223 336033).

### Reactions between CNA and diazafluorene: general procedure

A mixture of appropriate nitroalkene (**1a**–**1d** or **6**, 0.012 mol) and diazafluorene (0.010 mol) in 5 cm^3^ of the appropriate solvent was stirred at room temperature for 12 h. The solvent was evaporated in vacuo to dryness and the semisolid residue was recrystallized firstly from ethanol and then from cyclohexane.

#### *1*-*(4*-*Chlorophenyl)*-*2,3*-*diaza*-*4*-*(9*-*fluorenylidene)buta*-*1,3*-*diene* (**5a**, C_20_H_13_ClN_2_)

Yield: 95%; *t*_*R*_ = 10.6 min; yellow crystals; m.p.: 103–105 °C; IR (KBr): $\bar{\nu }$ = 2160, 1954, 1539, 1083 cm^−1^; ^1^H NMR (500 MHz, CDCl_3_): *δ* = 8.54 (s, 1H, CH), 7.92 (d, 1H, *J* = 7.6 Hz, CH_Ar_), 7.88 (d, 2H, *J* = 8.5 Hz, CH_Ar_), 7.68–7.62 (m, 2H, CH_Ar_), 7.52–7.43 (m, 5H, CH_Ar_), 7.35–7.31 (m, 2H, CH_Ar_) ppm; ^13^C NMR (125 MHz, CDCl_3_): *δ* = 158.2, 142.5, 137.3, 132.9, 131.6, 131.3, 131.0, 130.6, 129.8, 129.3, 128.3, 128.2, 123.0, 122.9, 120.1, 120.0, 119.9 ppm; MS: *m/z* = 316 (M^+^), 205; UV–Vis (methanol): *λ*_max_ = 342, 260, 196 nm.

#### *1*-*(4*-*Fluorophenyl)*-*2,3*-*diaza*-*4*-*(9*-*fluorenylidene)buta*-*1,3*-*diene* (**5b**, C_20_H_13_FN_2_)

Yield: 93%; *t*_*R*_ = 9.5 min; yellow crystals; m.p.: 99–101 °C; IR (KBr): $\bar{\nu }$ = 2160, 1957, 1549, 1103 cm^−1^; ^1^H NMR (500 MHz, CDCl_3_): *δ* = 8.56 (s, 1H, CH), 7.96 (dd, 2H, *J* = 5.5 Hz, 8.7 Hz, CH_Ar_), 7.92 (d, 1H, *J* = 7.6 Hz, CH_Ar_), 7.66–7.62 (m, 2H, CH_Ar_), 7.46–7.43 (m, 3H, CH_Ar_), 7.33 (q, 2H, *J* = 8.7 Hz, CH_Ar_), 7.22 (t, 2H, *J* = 8.5 Hz, CH_Ar_) ppm; ^13^C NMR (125 MHz, CDCl_3_): *δ* = 158.5, 142.1 (d, *J*_C–F_ = 114.4 Hz), 136.8, 131.7, 131.5, 131.2, 130.7, 130.6, 130.5, 130.3, 128.2, 122.9, 120.0, 119.9, 116.3, 116.1, 116.0 ppm; MS: *m/z* = 300 (M^+^), 205; UV–Vis (methanol): *λ*_max_ = 338, 259, 201 nm.

#### *1*-*Phenyl*-*2,3*-*diaza*-*4*-*(9*-*fluorenylidene)buta*-*1,3*-*diene* (**5c**, C_20_H_14_N_2_)

Yield: 95%; *t*_*R*_ = 8.5 min; orange crystals; m.p.: 83–84 °C; IR (KBr): $\bar{\nu }$ = 2161, 1955, 1543, 1099 cm^−1^; ^1^H NMR (500 MHz, CDCl_3_): *δ* = 8.59 (s, 1H, CH), 8.49 (d, 1H, *J* = 7.5 Hz, CH_Ar_), 7.97–7.93 (m, 3H, CH_Ar_), 7.66–7.62 (m, 2H, CH_Ar_), 7.54–7.52 (m, 3H, CH_Ar_), 7.46–7.42 (m, 2H, CH_Ar_), 7.36–7.31 (m, 2H, CH_Ar_) ppm; ^13^C NMR (125 MHz, CDCl_3_): *δ* = 160.4, 159.6, 142.5, 136.9, 134.4, 131.7, 131.5, 131.2, 130.7, 129.0, 128.7, 128.1, 127.9, 127.7, 125.5, 122.9, 120.8, 119.9 ppm; MS: *m/z* = 282 (M^+^), 205; UV–Vis (methanol): *λ*_max_ = 338, 255, 208 nm.

#### *1*-*(4*-*Methoxylphenyl)*-*2,3*-*diaza*-*4*-*(9*-*fluorenylidene)buta*-*1,3*-*diene* (**5d**, C_21_H_16_N_2_O)

Yield: 95%; *t*_*R*_ = 9.2 min; yellow crystals; m.p.: 127–130 °C; IR (KBr): $\bar{\nu }$  = 2160, 2038, 1599, 1099 cm^−1^; ^1^H NMR (500 MHz, CDCl_3_): *δ* = 8.58 (s, 1H, CH), 8.57 (d, 1H, *J* = 7.8 Hz, CH_Ar_), 7.94 (d, 1H, *J* = 9.5 Hz, CH_Ar_), 7.91 (d, 2H, *J* = 8.6 Hz, CH_Ar_), 7.65 (dd, 2H, *J* = 8.2 Hz, 5.2 Hz, CH_Ar_), 7.43 (t, 2H, *J* = 6.7 Hz, CH_Ar_), 7.35–7.31 (m, 2H, CH_Ar_), 7.05–7.03 (m, 2H, CH_Ar_), 3.90 (s, 3H, CH_3_) ppm; ^13^C NMR (125 MHz, CDCl_3_): *δ* = 162.3, 160.3, 160.0, 142.4, 137.0, 131.8, 131.3, 131.0, 130.8, 130.7, 130.5, 128.2, 128.1, 127.3, 122.8, 119.9, 119.8, 114.5, 55.5 ppm; MS: *m/z* = 312 (M^+^), 205; UV–Vis (methanol): *λ*_max_ = 358, 225, 197 nm.

#### *4′,5′*-*Dihydro*-*4′*-*nitro*-*5′*-*(trichloromethyl)spiro[9H*-*fluorene*-*9,3′*-*[3H]pyrazole]* (**7**, C_16_H_10_Cl_3_N_3_O_2_)

Yield: 93%; *t*_*R*_ = 14.8 min; white crystals; m.p.: 138–140 °C; IR (KBr): $\bar{\nu }$ = 2993, 2950, 1557, 1359, 748 cm^−1^; ^1^H NMR (500 MHz, CDCl_3_): *δ* = 7.83 (d, 1H, *J* = 8.5 Hz, CH_Ar_), 7.79 (d, 1H, *J* = 6.9 Hz, CH_Ar_), 7.61–7.58 (m, 1H, CH_Ar_), 7.52 (t, 1H, *J* = 7.6 Hz, CH_Ar_), 7.46–7.42 (m, 2H, CH_Ar_), 7.33–7.29 (m, 1H, CH_Ar_), 7.03 (d, 1H, *J* = 7.0 Hz, CH_Ar_), 6.82 (d, 1H, *J* = 7.6 Hz, CH_Ar_), 5.70 (d, 1H, *J* = 7.9 Hz, CH_Ar_) ppm; ^13^C NMR (125 MHz, CDCl_3_): *δ* = 142.0, 134.7, 131.5, 131.1, 129.0, 128.4, 125.2, 124.1, 121.0, 120.9, 104.5, 103.8, 94.8, 88.6 ppm.

#### *4′,5′*-*Dihydro*-*4′*-*nitro*-*5′*-*(dichloromethylene)spiro[9H*-*fluorene*-*9,3′*-*[3H]pyrazole]* (**8**, C_16_H_9_Cl_2_N_3_O_2_)

Yield: 96%; white crystals; m.p.: 175–177.5 °C; IR (KBr): $\bar{\nu }$ = 3014, 2975, 2910, 1629, 1560, 1353, 742 cm^−1^; ^1^H NMR (500 MHz, CDCl_3_): *δ* = 7.83–7.81 (m, 2H, CH_Ar_), 7.58–7.52 (m, 2H, CH_Ar_), 7.36–7.31 (m, 2H, CH_Ar_), 7.11 (d, 1H, *J* = 7.9 Hz, CH_Ar_), 6.86 (d, 1H, *J* = 8.6 Hz, CH_Ar_), 5.73 (s, 1H, CH_Ar_) ppm; ^13^C NMR (125 MHz, CDCl_3_): *δ* = 142.2, 140.5 136.4, 134.5, 131.3, 131.0, 129.0, 128.8, 124.9, 122.6, 121.1, 120.9, 101.2, 87.9 ppm; MS: *m/z* = 345 (M^+^).

### Quantum chemical calculations

All calculations reported in this paper were performed on “Zeus” supercomputer in the “Cyfronet” computational center in Cracow. Global and local electronic properties were estimated on the basis of structures obtained—according to Domingo suggestions [47, 48]—on the basis of B3LYP/6-31G(d) calculations. For this purpose, we have used structures created by the standard procedure [49]. In particular, the electronic chemical potentials (μ) and chemical hardness (*η*) were evaluated in terms of one-electron energies of FMO (*E*_HOMO_ and *E*_LUMO_) using the equations:

$$\mu \approx (E_{\text{HOMO}} +^{{}} E_{\text{LUMO}} )/2 \, \eta \approx E_{\text{LUMO}} - E_{\text{HOMO}} .$$

Next, the values of μ and η were then used for the calculation of global electrophilicity (ω) [47, 48] according to the formula:

$$\omega = \mu^{2} /2\eta ,$$

and the global nucleophilicity (*N*) [50] can be expressed in terms of the equation:

$$N = E_{\text{HOMO(diazafluorene)}} - E_{\text{HOMO (tetracyanoethene)}} .$$

The local nucleophilicity (*N*_*k*_) [51] condensed to atom *k* was calculated using global nucleophilicity *N* and Parr function P_k_^−^ [52] according to the formula:

$$N_{k} = \, P_{k}^{ - } N.$$

For the simulation of the reaction paths, hybrid functional B3LYP with the 6-31 ++G(d), basis set included in the GAUSSIAN 09 package [53] was used. It was found previously that calculations using B3LYP functional illustrate well the structure of transition states in polar 1,3-dipolar cycloadditions involving conjugated nitroalkenes [22, 23, 30]. The critical points on the reaction paths were localized in an analogous manner as in the case of the previously analyzed cycloadditions of diaryldiazomethanes with nitroacetylene [54]. In particular, for structure optimization of the reactants and the reaction products, the Berny algorithm was applied. First-order saddle points were localized using the QST2 procedure. The transition states were verified by diagonalization of the Hessian matrix and by an analysis of the intrinsic reaction coordinates (IRC).

All calculations were carried out for the simulated presence of nitromethane as the reaction medium. For this purpose, PCM [55] was used. For optimized structures, the thermochemical data for the temperature *T* = 298 K and pressure *p* = 1 atm were computed using vibrational analysis data. Global electron density transfer (GEDT) [56] was calculated according to the formula:

$$GEDT \, = \, - \varSigma q_{A} ,$$

where *q*_*A*_ is the net Mulliken charge and the sum is taken over all the atoms of the dipolarophile. Indexes of σ-bonds development (*l*) were calculated according to the formula [30]:

$${l_{A - B} = 1 - \frac{{r_{A - B}^{TS} - r_{A - B}^{P} }}{{r_{A - B}^{P} }}},$$

where *r*_*A*–*B*_^*TS*^ is the distance between the reaction centers A and B at the TS and *r*_*A*–*B*_^*P*^ is the same distance at the corresponding product.

## Electronic supplementary material

Below is the link to the electronic supplementary material.

Supplementary material 1 (DOC 989 kb)
