# Supplementary material for: Unexpected course of reaction between (E)-2-aryl-1-cyano-1-nitroethenes and diazafluorene: why is there no 1,3-dipolar cycloaddition?
Source: Monatsh Chem. 2017 Mar 22;148(5):909–15. doi: 10.1007/s00706-016-1893-5 (PMC5387018; doi:10.1007/s00706-016-1893-5)

Supplementary Material

| Unexpected course of reaction between  (E)-2-aryl-1-cyano-1-nitroethenes and diazafluorene:  why is there no 1,3-dipolar cycloaddition?  Radomir Jasiński ● Karolina Kula ● Agnieszka Kącka ● Barbara Mirosław |
| --- |

**PHYSICAL CHARACTERISTICS**

*1-(4-chlorophenyl)-2,3-diaza-4-(9-fluorenylidene)-buta-1,3-diene*

**^1^H MNR**


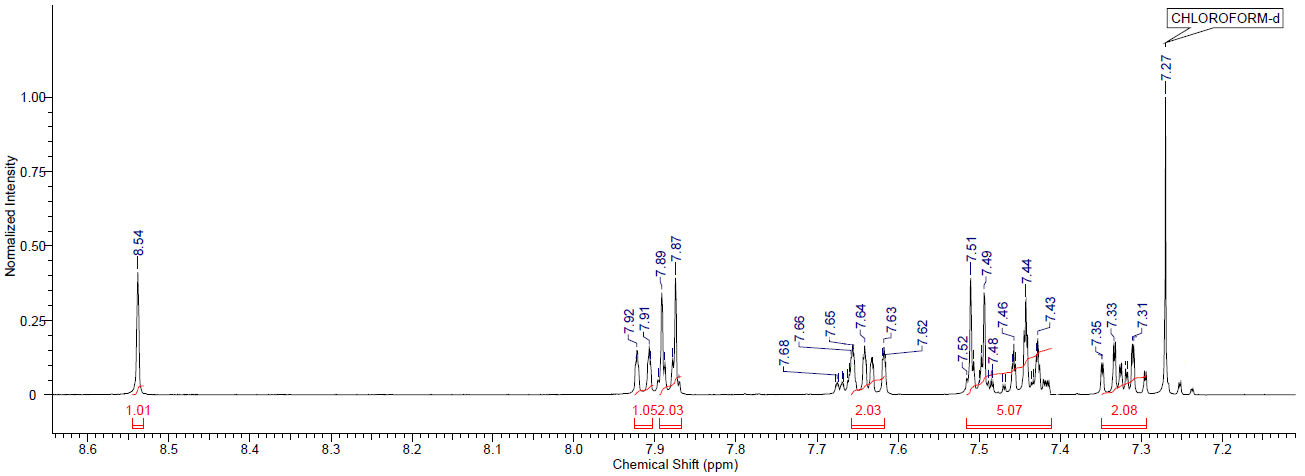


**^13^C NMR**


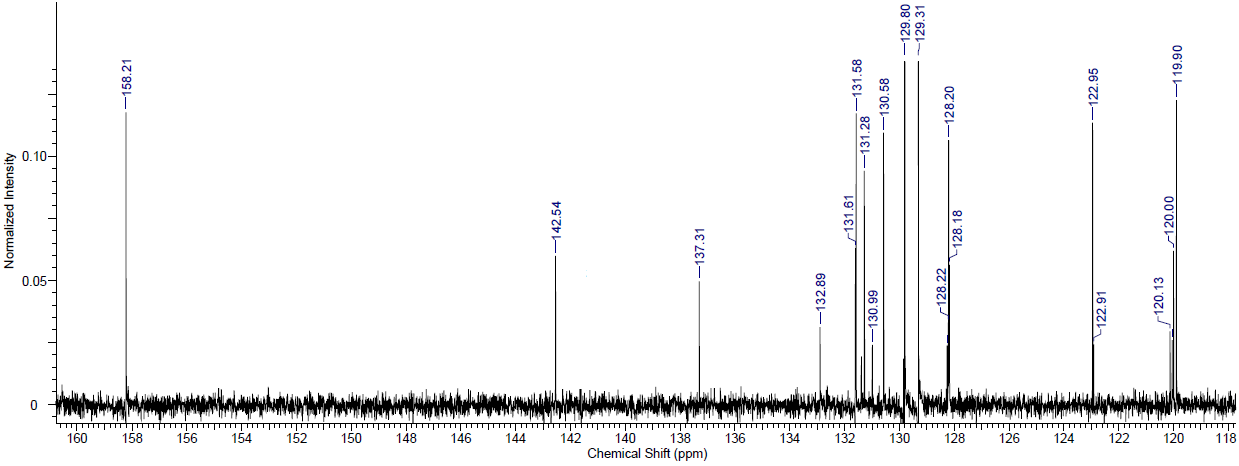


**X-RAY structural analysis**

Molecular structure of with atom labels:


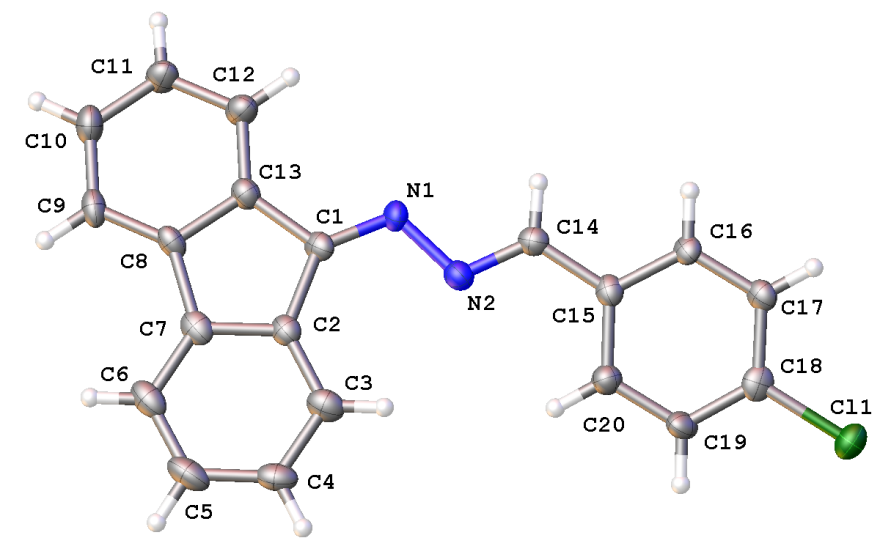


Crystal data and structure refinement:

| Empirical formula | C_20_H_13_N_2_Cl |
| --- | --- |
| Formula weight | 316.79 |
| Temperature/K | 120.0(1) |
| Crystal system | Orthorhombic |
| Space group | *Pna*2_1_ |
| a/Å | 17.1599(18) |
| b/Å | 15.0993(19) |
| c/Å | 5.7829(6) |
| Volume/Å^3^ | 1498.4(3) |
| Z | 4 |
| ρ_calc_g/cm^3^ | 1.4042 |
| μ/mm^‑1^ | 2.241 |
| F(000) | 656.2 |
| 2Θ range for data collection/° | 7.8 to 153.64 |
| Reflections collected | 5322 |
| Independent reflections | 2239 [*R*_int_ = 0.0794, *R*_sigma_ = 0.0781] |
| Data/parameters | 2239/207 |
| Goodness-of-fit on *F*^2^ | 1.040 |
| Final *R* indexes [I>=2σ (I)] | *R*_1_ = 0.0566, w*R*_2_ = 0.1478 |
| Final *R* indexes [all data] | *R*_1_ = 0.0684, w*R*_2_ = 0.1675 |
| Largest diff. peak/hole / e Å^-3^ | 0.44/-0.61 |
| Flack parameter | 0.02(4) |
| CCDC No. | 1448932 |

*1-(4-fluorophenyl)-2,3-diaza-4-(9-fluorenylidene)-buta-1,3-diene*

**^1^H MNR**


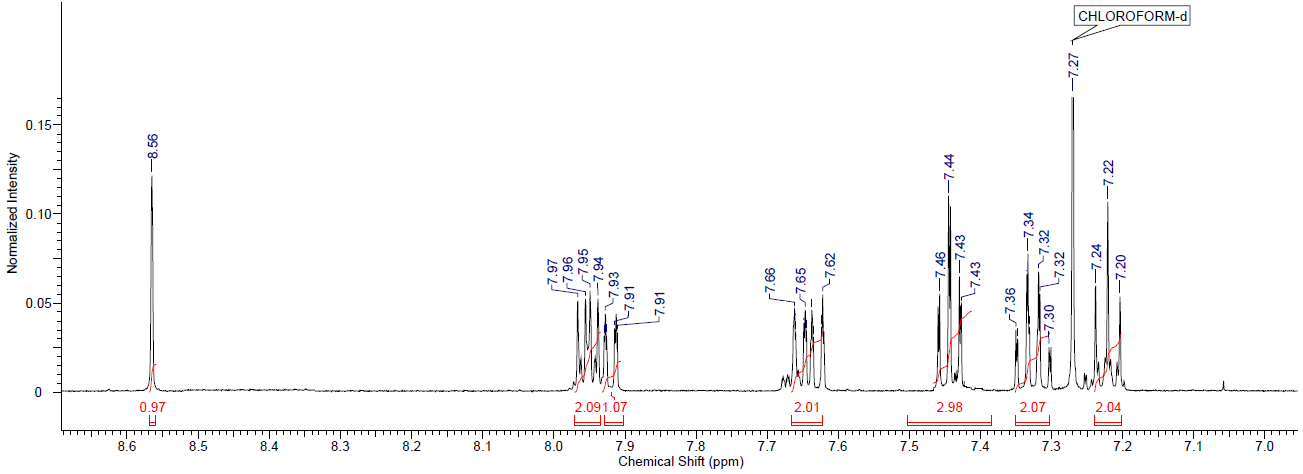


**^13^C NMR**


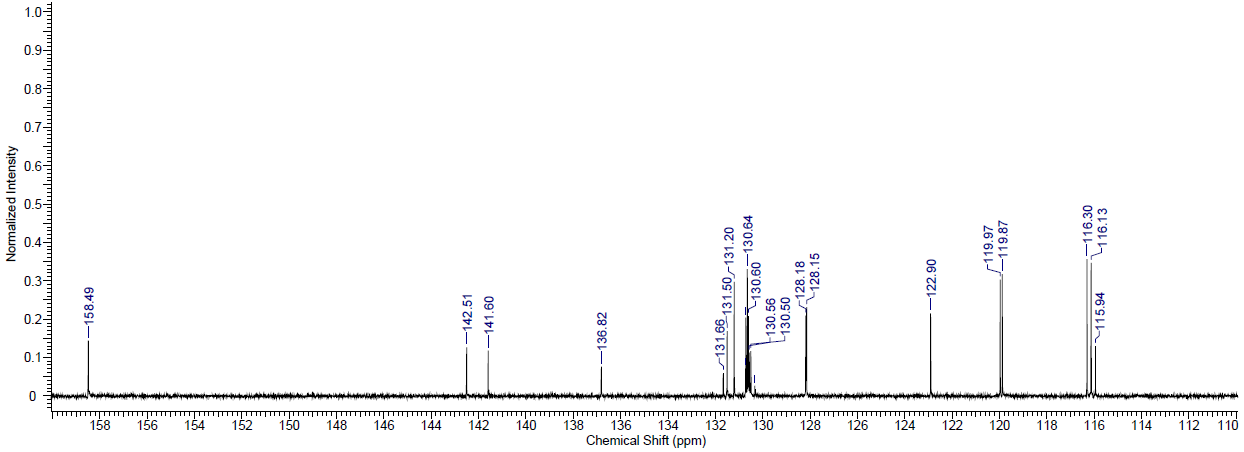


**^19^F NMR**


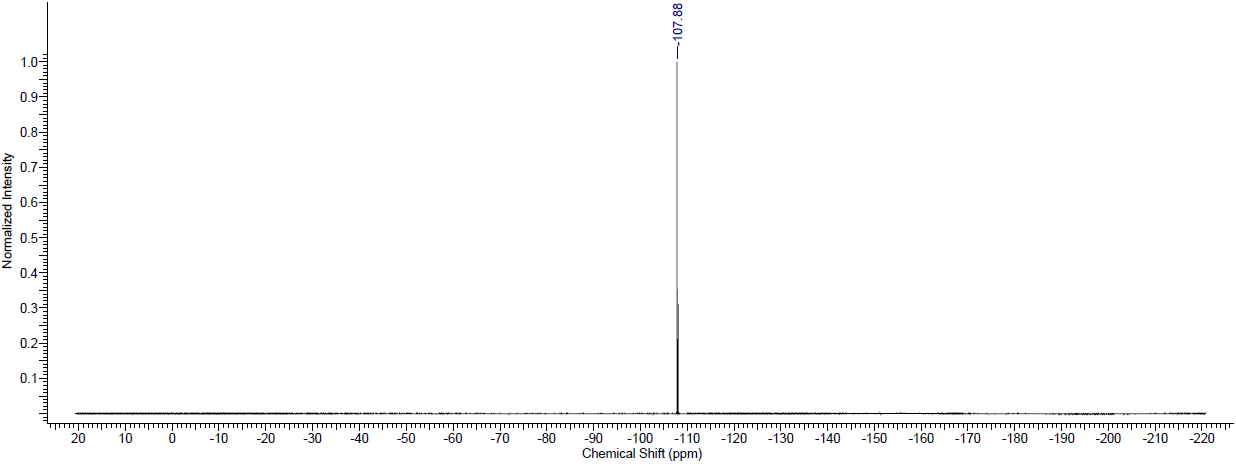


*1-phenyl-2,3-diaza-4-(9-fluorenylidene)-buta-1,3-diene*

**^1^H MNR**


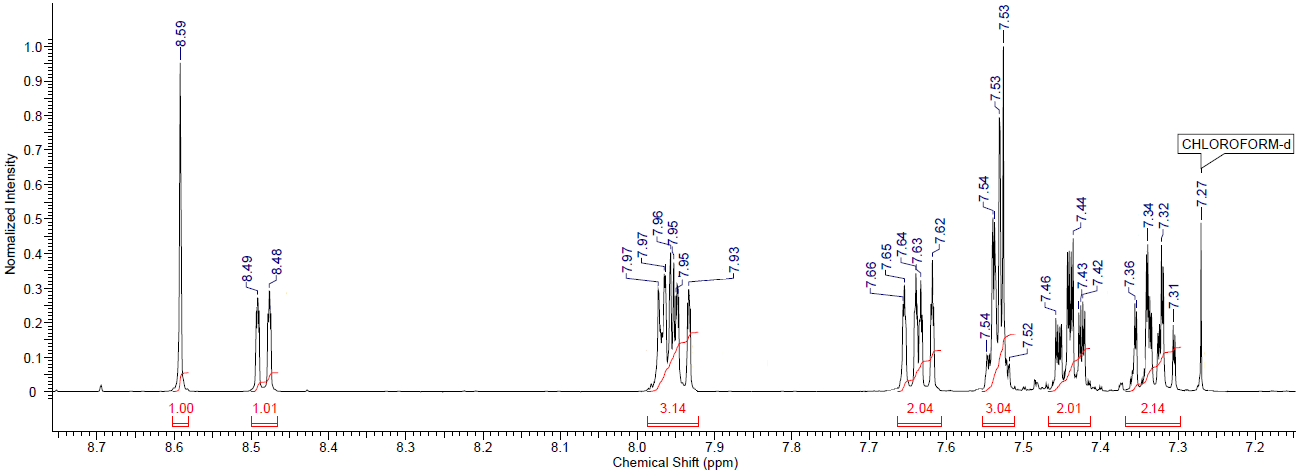


**^13^C NMR**


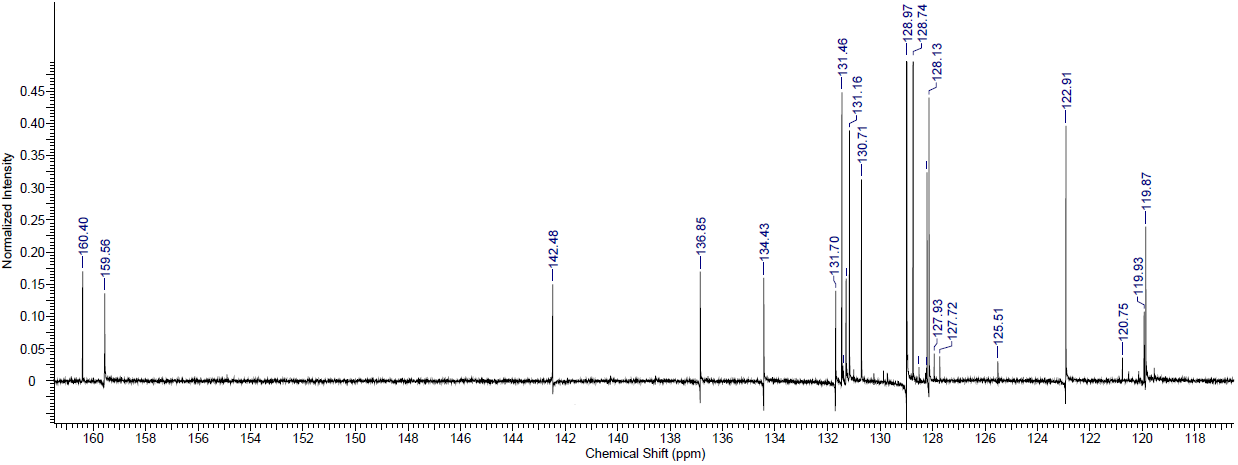


*1-(4-methoxylphenyl)-2,3-diaza-4-(9-fluorenylidene)-buta-1,3-diene*

**^1^H MNR**


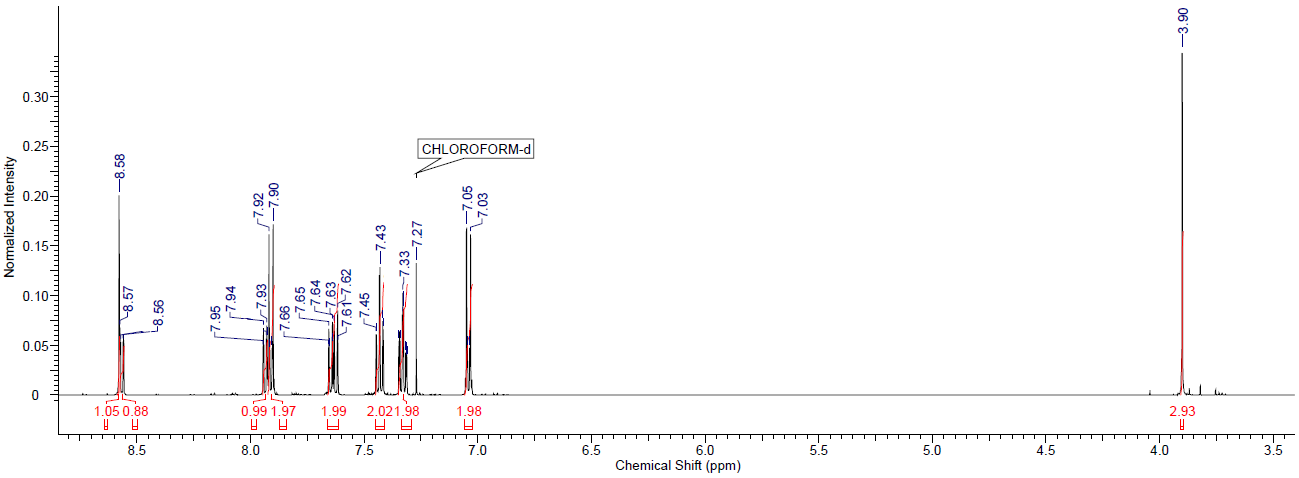


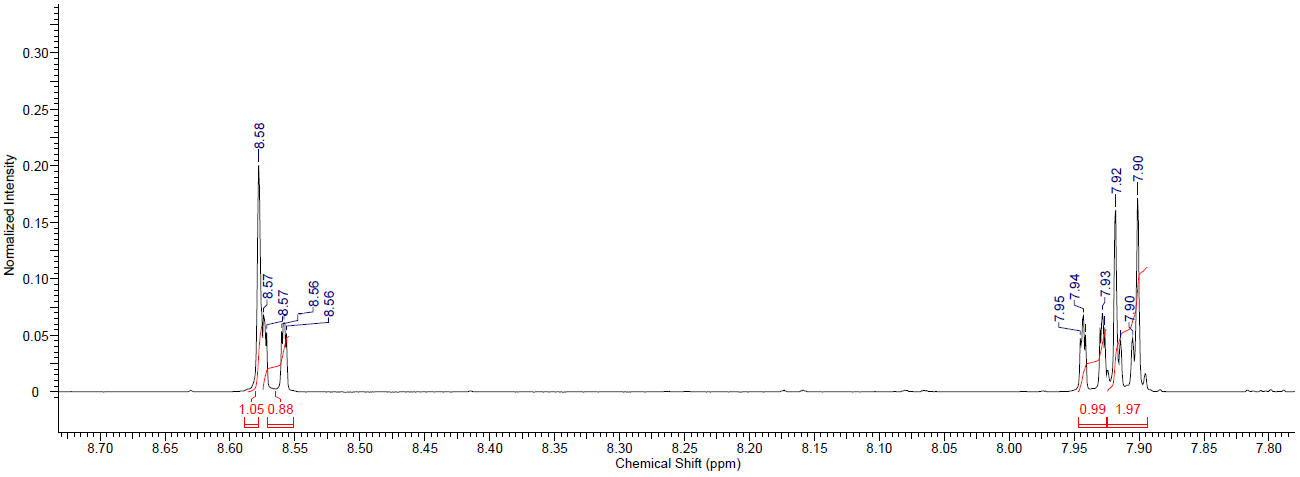


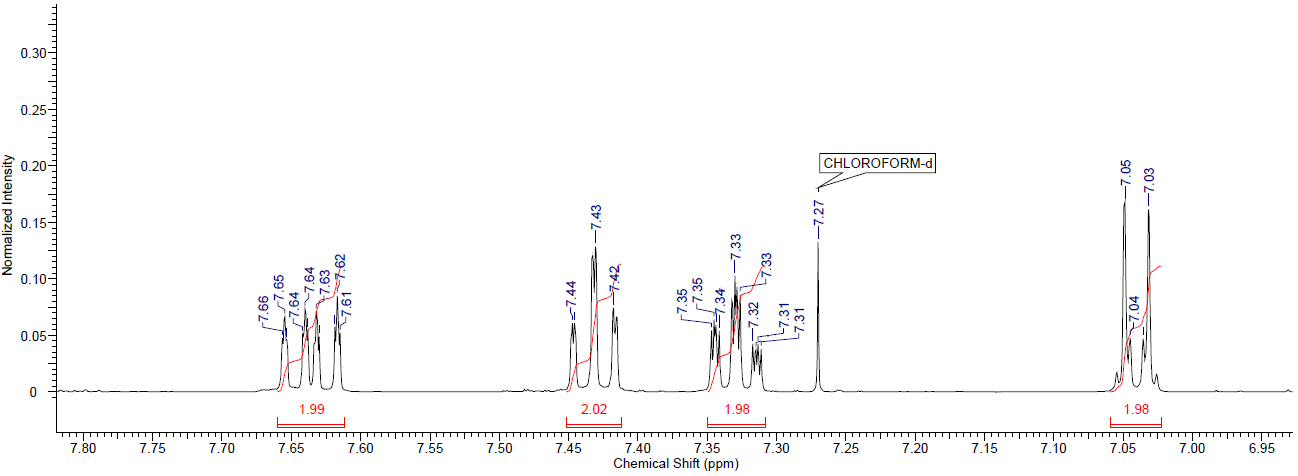


**^13^C NMR**


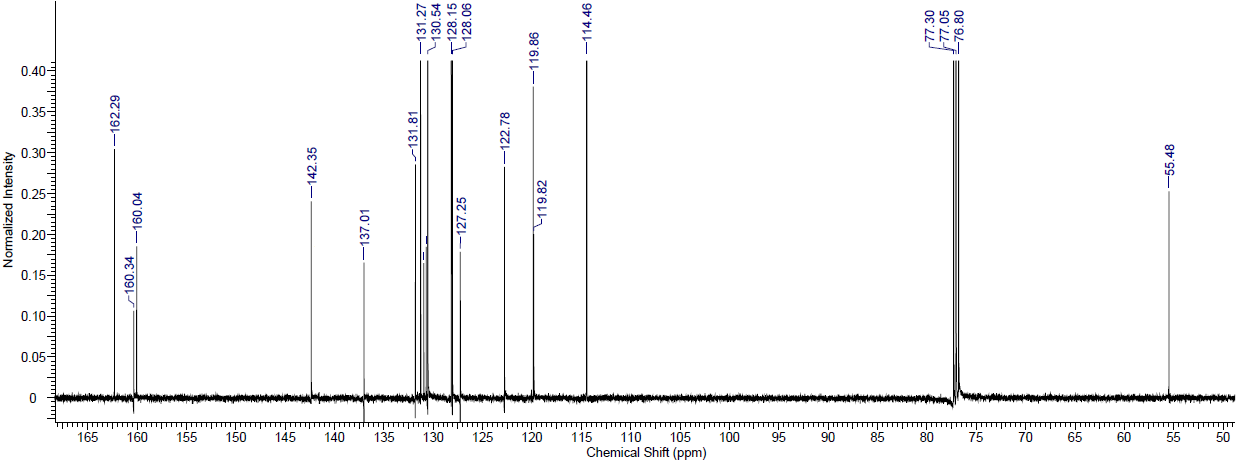


*3-spiro(dibenzene-cyclopentyl)-4-nitro-5-trichloromethyl-Δ^1^-pyrazoline*

**^1^H NMR**

**
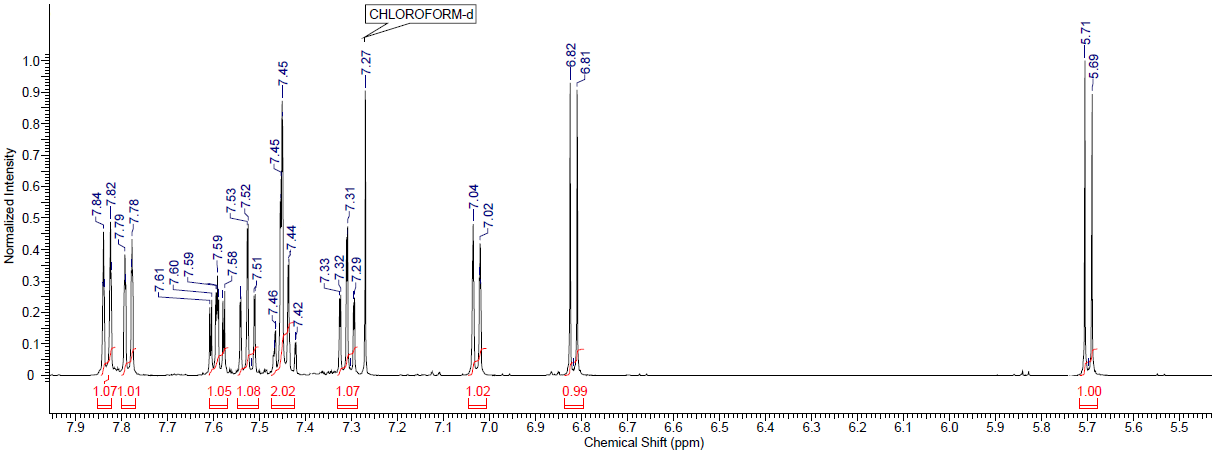
**

**^13^C NMR**

**
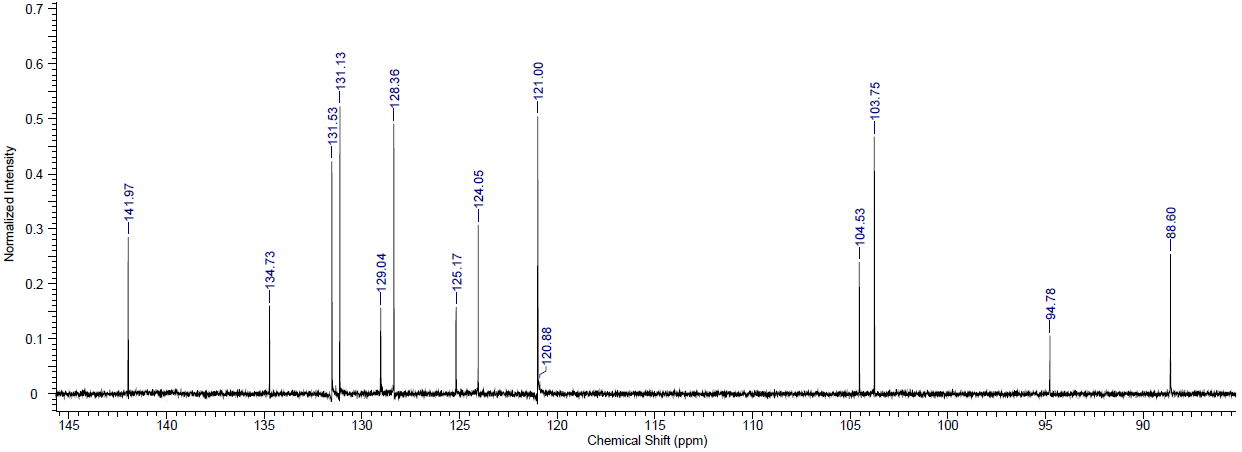
**

*3-spiro(dibenzene-cyclopentyl)-4-nitro-5-dichloromethylidene-Δ^1^-pyrazoline*

**^1^H NMR**


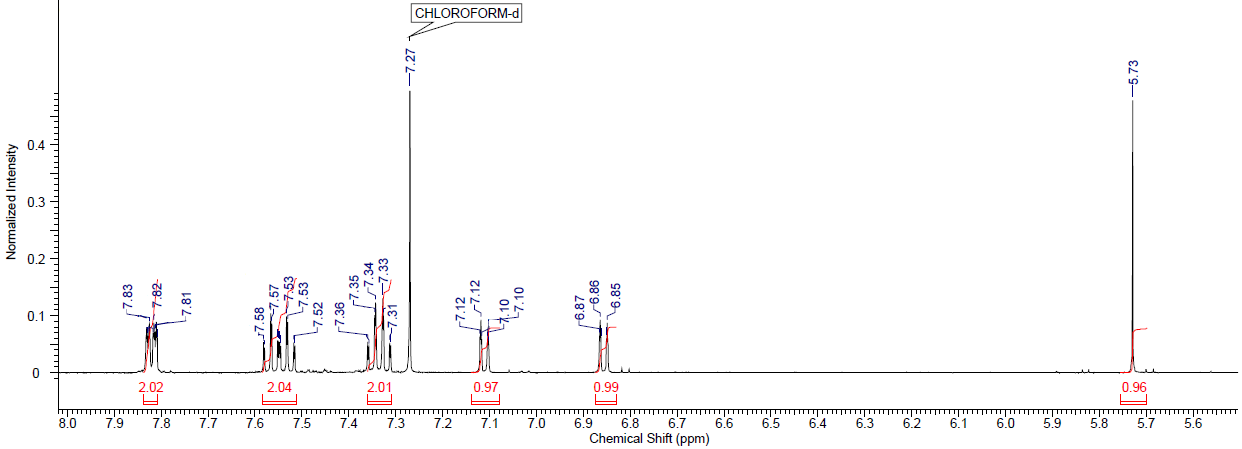


**^13^C NMR**


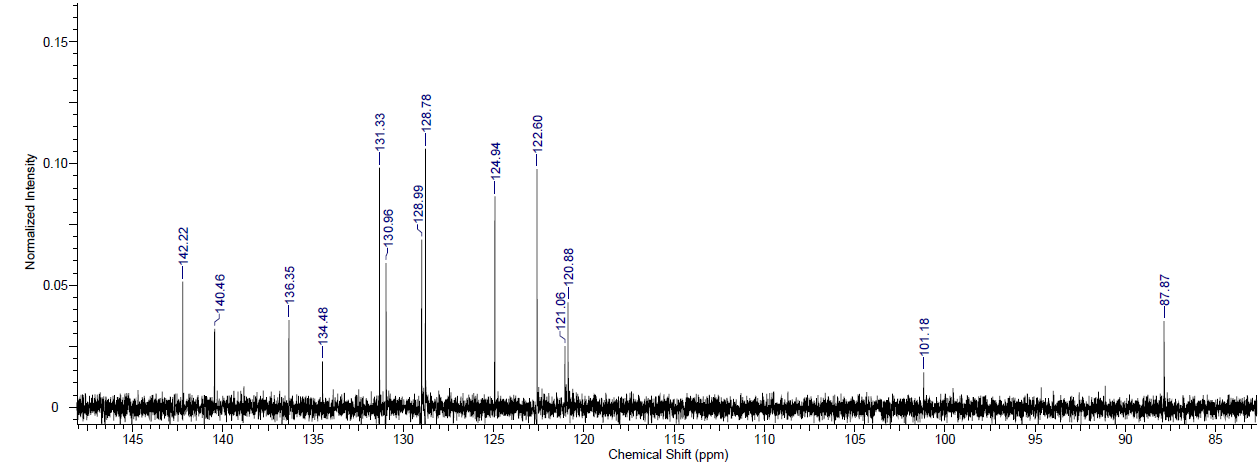

Supplement: Supplementary file 1 — Supplementary material 1 (DOC 989 kb) [file 706_2016_1893_MOESM1_ESM.doc]
